# Associations between spike velocity and physical characteristics in elite sitting volleyball players

**DOI:** 10.3389/fphys.2026.1992570

**Published:** 2026-10-12

**Authors:** Shaocheng Sun, Meiling Ren, Jinhu Yan

**Affiliations:** 1 School of Physical Education & Health, Shanghai University of International Business and Economics, Shanghai, China; 2 The Branch of Beijing No.2 Middle School, Beijing, China

**Keywords:** anaerobic power, anthropometry, para-athletes, physical fitness, sitting volleyball, spike velocity, upper-body strength

## Abstract

**Purpose:**

This study examined associations between spike velocity and anthropometric characteristics, anaerobic performance, and physical fitness in elite sitting volleyball players.

**Methods:**

Thirty elite sitting volleyball players performed six maximal-effort spike trials and completed assessments of seated anthropometry, arm-crank anaerobic performance, upper-body strength and power, trunk function, and sport-specific movement performance. Pooled Pearson correlations, sex-adjusted partial correlations, sex-specific descriptive analyses, exploratory sex-adjusted regression, and a VS1-only sensitivity analysis were performed. Between-sex comparisons and correlation analyses used Benjamini–Hochberg (BH) false discovery rate (FDR) correction.

**Results:**

Men achieved higher spike velocity than women (67.22 ± 6.74 vs. 51.51 ± 6.04 km/h; Hedges’ *g* = 2.38, BH-adjusted *p* < 0.001). In the pooled sample, spike velocity showed strong positive correlations with seated vertical grip reach (*r* = 0.80), sitting height (*r* = 0.76), handgrip strength (*r* = 0.75), one-repetition maximum bench press (*r* = 0.74), and seated medicine-ball chest-pass distance (*r* = 0.70), and moderate positive correlations with hand span (*r* = 0.69), seated rotational medicine-ball throw (*r* = 0.65), peak power (*r* = 0.51), and mean power (*r* = 0.51). After adjustment for sex, most of the positive pooled associations were substantially attenuated, and none remained significant after FDR correction. The largest absolute sex-adjusted partial correlations were observed for hand span (partial *r* = 0.52), seated vertical grip reach (partial *r* = 0.48), relative peak power (partial *r* = −0.48), and sitting height (partial *r* = 0.40). Sex-specific descriptive analyses showed positive anthropometric correlations in both men and women, whereas strength and absolute anaerobic-power associations were less consistent. The exploratory regression including sex, seated vertical grip reach, and seated rotational medicine-ball throw accounted for 71.9% of the variance in spike velocity in this sample (adjusted *R*² = 0.687), with seated vertical grip reach retaining a positive coefficient (*B* = 0.457, *p* = 0.010). Excluding three VS2 athletes did not materially alter the findings.

**Conclusion:**

Several strong pooled associations were substantially influenced by between-sex differences. Seated anthropometric measures showed the most consistent positive associations across adjusted and sex-specific analyses, but the findings remain associative and exploratory.

## 1 Introduction

Sitting volleyball is a Paralympic net sport in which athletes perform volleyball-specific technical and tactical actions while moving across the court in a seated position. Under the official rules, restrictions on lifting the body from the court while playing the ball distinguish sitting volleyball from its standing counterpart (World ParaVolley, 2025). Because the conventional standing approach and jump are absent, lower-limb propulsion contributes less, placing greater demands on seated positioning, floor-based movement, trunk control, and upper-extremity function. Athletes with eligible physical impairments are assigned to one of two sport classes according to the impact of their impairment on activities fundamental to sitting volleyball. VS1 comprises athletes whose impairments have a greater impact on these core functions, whereas VS2 comprises athletes who meet the minimum impairment criteria but whose impairments have a smaller impact on these functions (World ParaVolley, 2026). Both classes compete together, with no more than one VS2 athlete permitted on court per team at a time. These sport classes describe impairment-related activity limitations rather than levels of playing ability.

The potential performance implications of these sport classes have received increasing research attention. Recent expert consensus on evidence-based classification in sitting volleyball has identified fundamental sport-specific skills as important performance determinants and highlighted upper-limb function as particularly relevant across volleyball activities, while emphasizing the need for further empirical evidence supporting sport-class criteria (Marszałek et al., 2024). Video analysis of seven fundamental sitting-volleyball skills in athletes from the Rio 2016 Paralympic Games found no overall difference in aggregate skill efficacy between the former disabled (D) and minimally disabled (MD) classes, although sex- and skill-specific differences were observed; these historical classes should not be assumed to correspond directly to the current VS1/VS2 system (Esatbeyoglu et al., 2021). Consequently, anthropometric and physical performance characteristics may interact with impairment-related functional limitations to influence sport-specific performance, and physical profiles may vary by competitive level and sex (Molik et al., 2017; Ahmadi et al., 2019, 2020; Cavedon et al., 2022; Petrigna et al., 2023).

Attacking effectiveness is central to success in elite sitting volleyball. Match analyses indicate that attack effectiveness and other offensive actions distinguish winning from losing teams, whereas seated vertical reach and impairment-related functional characteristics may influence game performance (Molik et al., 2017; Marszałek et al., 2018). The spike is the principal forceful attacking action and is intended to direct the ball rapidly and accurately into the opponent’s court. Although attack success also depends on set quality, attack direction, block organization, ball placement, and tactical decision-making, ball velocity is an objective and readily quantifiable component of spike performance. Greater velocity may reduce the time available for opposing blockers and floor defenders to perceive the ball trajectory and organize an effective response. Evidence from standing volleyball further shows that the spike involves high shoulder and elbow angular velocities, forces, and torques and that arm-swing technique and ball-to-hand contact influence the mechanics of the action (Coleman et al., 1993; Forthomme et al., 2005; Reeser et al., 2010; Baena-Raya et al., 2021; Giatsis and Tilp, 2022; Howard et al., 2023).

The volleyball spike belongs to a broader family of overhand striking and throwing actions that includes the volleyball serve, tennis serve, team-handball throw, and baseball pitch. These actions generate high distal-segment and ball velocities through coordinated multisegmental motion rather than the isolated action of a single joint or muscle group. A broadly proximal-to-distal organization has been described, in which motion is transferred from the trunk and proximal upper-limb segments toward the forearm and hand, although the precise sequence depends on the task and on how joint and segmental events are defined (Putnam, 1993; van den Tillaar and Ettema, 2009). Direct comparisons of the team-handball throw, tennis serve, and volleyball spike have identified shared upper-body coordination patterns alongside task-specific differences in trunk, shoulder, elbow, and wrist kinematics (Wagner et al., 2014). Baseball pitching research likewise indicates that pelvis and upper-torso orientation, rotational velocity, and the timing of trunk rotation are associated with ball velocity and upper-extremity loading (Stodden et al., 2001; Oyama et al., 2014). In sitting volleyball, restricted lower-limb energy generation may increase the relative importance of initiating and transferring motion through the trunk and upper extremities.

Anthropometric characteristics may shape the spatial and mechanical conditions under which a sitting volleyball spike is performed. Sitting height and seated vertical grip reach may influence attainable contact height, the ability to strike above the net, and the effective path over which the striking arm can accelerate. Hand span may also affect hand–ball interaction and ball control, although its direct contribution to spike velocity remains unclear. Previous sitting volleyball research has shown that arm reach and the heights attained in block and spike positions are related to anaerobic and field-test performance, whereas seated vertical reach has been associated with game performance and sport-specific floor movement (Marszałek et al., 2015; Molik et al., 2017; Cavedon et al., 2022). More directly, greater vertical reach facilitated a higher ball-impact position during the sitting volleyball serve, and impact height contributed to serve velocity (Cavedon et al., 2023). Tennis research similarly indicates that body height, sitting height, arm dimensions, body mass, and contact height may be associated with serve velocity, although the relevant predictors vary by sex, age, and playing level (Bonato et al., 2015; Fett et al., 2020; Hayes et al., 2021; Sánchez-Pay et al., 2021; Baiget et al., 2023). These findings support examining seated reach dimensions and hand characteristics in relation to spike velocity while recognizing that anthropometry may act partly through contact position and technique.

Upper-body strength and power may provide the force-generating foundation for a high-velocity spike. In 16 elite male national-team sitting volleyball players, Fashi et al. (2025) reported greater dominant-shoulder internal-rotator than external-rotator peak torque, total work, and average power during concentric isokinetic testing. This sport-specific evidence characterizes shoulder capacity, although it does not establish an association with spike velocity. In standing volleyball players, spike velocity has been associated with the concentric strength of the dominant shoulder internal rotators and elbow flexors and extensors, highlighting the relevance of the muscles that directly accelerate the striking arm (Forthomme et al., 2005). Greater muscular strength may improve force–time characteristics and provide a larger force reserve for explosive sporting actions (Suchomel et al., 2016). Force–velocity characteristics derived from ballistic bench-press testing and other performance profiles have also been associated with spike and serve velocity in top-level volleyball players (Baena-Raya et al., 2021; Terol-Sanchis et al., 2021). Team-handball research likewise links overarm throwing velocity with one-repetition maximum (1RM) bench press, bar velocity, peak power, fat-free mass, and upper-body power (Gorostiaga et al., 2005; Marques et al., 2007). However, longitudinal observations in professional female volleyball players indicate that changes in general strength and medicine-ball throwing performance do not necessarily correspond to changes in spike velocity (Valadés et al., 2016). Handgrip strength, 1RM bench press, and seated medicine-ball chest-pass performance may therefore provide complementary indicators of general, maximal, and explosive upper-body capacity, but none reproduces the unilateral, high-velocity coordination demands of the spike.

Trunk function may be particularly relevant in sitting volleyball because the trunk contributes to postural control, body positioning, rotation, and the transfer of motion toward the striking arm. Research on overhand striking and throwing emphasizes the importance of coordinated trunk and upper-limb sequencing for generating distal-segment velocity (Putnam, 1993; Stodden et al., 2001; Oyama et al., 2014; Wagner et al., 2014). However, field tests capture different aspects of trunk function. Curl-up and back-extension tests primarily assess dynamic muscular endurance, whereas a seated rotational medicine-ball throw requires rapid trunk rotation coordinated with arm movement. Side medicine-ball throw performance has been associated with trunk and whole-body physical capacities in male and female athletes (Ikeda et al., 2007), and seated rotational mobility and power have been linked to overarm throwing velocity (Talukdar et al., 2015). More recently, rotational medicine-ball throw performance has been associated with measured trunk-rotator strength, supporting its use as a functional indicator of rotational capacity (Rodriguez-Perea et al., 2024). Nevertheless, throwing distance is also influenced by release velocity, release angle, body dimensions, and technique and should not be interpreted as a direct measure of muscular power.

Anaerobic performance and sport-specific movement ability represent additional dimensions of the sitting volleyball physical profile. The arm-crank Wingate Anaerobic Test (WAnT) assesses the capacity to generate and sustain high upper-body power, whereas short sprints, multidirectional movement tests, and speed-endurance tasks assess the ability to reposition rapidly on court. In elite sitting volleyball players, WAnT outcomes have been associated with short-distance sprinting, agility, speed endurance, seated medicine-ball chest-pass performance, and selected game-performance measures (Marszałek et al., 2015). Field-fitness measures have also been associated with coach-rated sitting volleyball performance (Jeoung, 2017), and male and female national-team players have shown substantial differences in power- and speed-oriented field tests (Ahmadi et al., 2019). Comparisons between national- and league-level players further suggest that physical-test profiles reflect competitive level (Ahmadi et al., 2020), whereas anthropometric and physical fitness characteristics have been related to floor-based sprint performance (Cavedon et al., 2022). These findings support the relevance of anaerobic and movement capacities to overall sitting volleyball performance. However, their relationships with the velocity of a single standardized maximal spike remain uncertain because the tests differ in duration, movement pattern, and technical specificity.

Despite growing interest in sitting volleyball performance, the available evidence remains limited and methodologically heterogeneous. A scoping review identified only a small number of studies assessing physical fitness in sitting volleyball and highlighted substantial variation in testing protocols and outcome measures (Petrigna et al., 2023). Existing studies have primarily examined anthropometric profiles, classification, field-test performance, game efficiency, competitive level, or serve velocity (Marszałek et al., 2015, 2024; Jeoung, 2017; Molik et al., 2017; Ahmadi et al., 2020; Esatbeyoglu et al., 2021; Cavedon et al., 2022, 2023). Direct radar-based measurement of spike velocity has also been reported in 13 elite male national-team players in a randomized crossover study of acute caffeine supplementation (Diedhiou et al., 2025). Evidence relating spike velocity to athletes’ anthropometric and physical-performance characteristics remains limited, particularly across multiple physical domains in both men and women. Therefore, this study aimed to examine the associations between spike velocity and anthropometric characteristics, anaerobic performance, and physical fitness in elite sitting volleyball players. We hypothesized that greater seated reach dimensions, upper-body strength and power, rotational throwing performance, and absolute anaerobic power would be associated with greater spike velocity, whereas shorter completion times in sport-specific movement tests would be associated with greater spike velocity.

## 2 Methods

### 2.1 Participants

Thirty elite sitting volleyball players from the Chinese men’s and women’s national teams were recruited during a national-team training camp before the 2026 Sitting Volleyball World Championships in Hangzhou, China. The sample included 16 men (age: 29.15 ± 7.73 years; training experience: 11.25 ± 6.92 years) and 14 women (age: 34.16 ± 6.89 years; training experience: 14.25 ± 6.45 years). The athletes’ recorded World ParaVolley sport classes were VS1 for 27 participants and VS2 for three participants. To protect confidentiality in this small national-team cohort, impairment characteristics were summarized at group level. Among the 27 VS1 athletes, 13 were prosthesis users, whereas the remaining 14 had non-amputation impairments characterized by preserved anatomical structure but functional limitations. According to the available classification records, the three VS2 athletes had non-amputation lower-limb impairments classified as impaired passive range of movement (PROM): one primarily involved the ankle and two primarily involved the hip; none used a prosthesis. Individual diagnoses are not reported. For the purposes of this study, “elite” was operationally defined as current membership of the Chinese national team together with at least three years of international competition experience. The required sample size was estimated *a priori* using G*Power software (version 3.1) with the “Exact tests—Correlation: Difference from constant (one sample), bivariate normal model” procedure (two-tailed; ρ H0 = 0; ρ H1 = 0.45; α = 0.05; power = 0.80). Under these settings, the minimum required sample size was 35 participants. Owing to the limited availability of eligible elite national-team sitting volleyball players, 30 athletes were ultimately recruited and completed the study. All participants provided written informed consent before participation. The study was approved by the Beijing Sport University Human Participants Review Board (no. 2025195H) and conducted in accordance with the Declaration of Helsinki.

### 2.2 Design and procedures

Participants completed one familiarization session and three testing sessions at the Shanghai Special Olympics Competition and Training Center on four separate days, with 48 h between consecutive sessions. All sessions were conducted at approximately the same time of day within a 2-h window. Participants were instructed to refrain from strenuous physical activity for 48 h before each session and to maintain their usual diet and hydration habits. Before each testing session, participants completed a standardized warm-up consisting of specific mobility exercises and dynamic stretching. Assessments were performed in the order presented below and arranged in accordance with National Strength and Conditioning Association (NSCA) recommendations to minimize the potential influence of fatigue on subsequent tests (Haff and Triplett, 2016). A rest interval of 2–5 min was provided between tests. During the first testing session, participants completed the anthropometric assessments, handgrip strength test, 1RM bench press test, seated medicine-ball chest pass, and seated rotational medicine-ball throw. During the second testing session, they completed the spike velocity test, 5-m forward and backward sprint tests, modified agility T-test, speed-endurance test, curl-up test, and back-extension endurance test. During the third testing session, participants completed the 30-s WAnT. No class-specific test modifications were made; when required, only individual positioning or stabilization was adapted, while the testing and scoring procedures remained unchanged.

#### 2.2.1 Spike velocity test

Spike velocity was measured using a radar gun (Speedster II; Bushnell, Overland Park, KS, USA). Ball model (V5M5000; Molten Corporation, Hiroshima, Japan) and inflation pressure were standardized across trials. The net height followed official international sitting volleyball rules: 1.15 m for men and 1.05 m for women (World ParaVolley, 2025). After three submaximal practice spikes, each participant performed six maximal-effort valid spike trials, each comprising a preparatory movement followed by an overhand striking action (Forthomme et al., 2005; Valadés et al., 2016). A 1-min rest interval was provided between trials. The same experienced setter was used throughout testing and delivered the ball from the predefined setter starting zone while attempting to reproduce a consistent set trajectory. Set height and set position were not instrumentally measured. Participants were instructed to direct each spike toward a predefined diagonal target zone (**Figure 1**). A valid trial required a set within the intended hitting area, a legal overhand spike crossing the net into the predefined target zone, and a radar recording without evident measurement interference. Attempts not meeting these criteria were excluded and repeated until six valid trials were completed. Following previously described radar-based ball-speed procedures (Cavedon et al., 2023; Diedhiou et al., 2025), a single experienced operator positioned the radar along the extension of the expected post-impact ball-flight path. Owing to the dimensions of the testing venue, the placement distance was adapted to 3.0 m from the net. The radar axis was aligned as parallel as possible to the ball trajectory at approximately the height of ball contact to minimize cosine error; the alignment angle was not instrumentally quantified. While the trigger was activated, the radar gun continuously tracked ball velocity, and the highest velocity detected during each valid trial was recorded. The highest velocity across the six valid trials was retained as the measure of maximal spike performance because the study aimed to characterize maximal spike-velocity capacity; six valid attempts provided repeated opportunities to achieve a representative maximal effort while the 1-min intertrial rest limited fatigue. The retained value was expressed in kilometers per hour (km/h). Diedhiou et al. (2025) similarly used radar-based spike testing with a best-trial outcome in elite sitting volleyball players, although their protocol comprised three attempts. Within-session consistency across the six valid attempts was quantified using a two-way mixed-effects, absolute-agreement intraclass correlation coefficient (ICC) and the coefficient of variation (CV).

**Figure 1:**
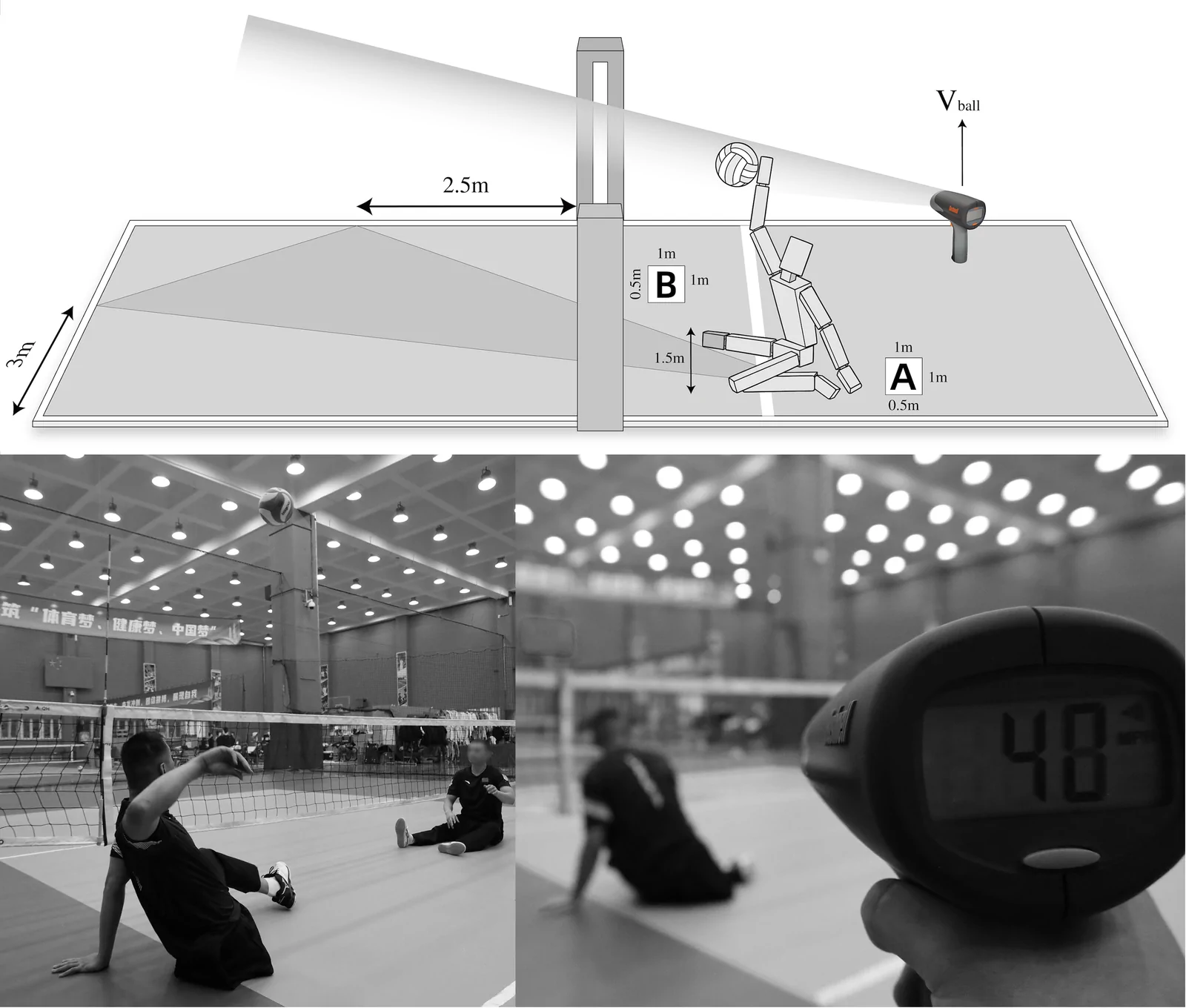
Experimental setup for the spike velocity test. **(A)** Starting zone of the spiker 0.50 m from the left side and 2.50 m from the central line; **(B)** starting zone of the setter 3.50 m from the left side and 0.50 m from the central line.

#### 2.2.2 Anthropometric assessment

Anthropometric measurements were obtained by an operator certified by the International Society for the Advancement of Kinanthropometry (ISAK) in accordance with the International Standards for Anthropometric Assessment (Esparza-Ros et al., 2019), with impairment-specific adaptations for participants with motor disabilities where necessary (Broad et al., 2026). Body mass was measured using a calibrated wheelchair scale (Seca 677; Seca GmbH & Co. KG, Hamburg, Germany). Net body mass, excluding prosthesis mass, was used to calculate BMI and to normalize Wingate loading and power. Standing height was obtained in all participants using a measuring rod (Seca 220; Seca GmbH & Co. KG, Hamburg, Germany); when conventional unsupported standing was not feasible, positioning or stabilization assistance was used while maintaining the standard anatomical landmarks as closely as possible in accordance with motor-disability adaptations. Body mass index (BMI) was calculated as body mass divided by height squared (kg/m²) and was treated as a descriptive anthropometric index because conventional BMI may not be directly comparable across impairment types, particularly in athletes with lower-limb amputation. To reflect the sport-specific playing posture, seated anthropometric measurements were obtained with participants seated on the floor and their lower limbs extended (Cavedon et al., 2015, 2018). Sitting height and seated vertical grip reach were measured using a Harpenden anthropometer (Holtain Ltd., Crymych, Pembrokeshire, UK). Sitting height was defined as the vertical distance from the vertex of the head to the floor. Seated vertical grip reach was defined as the maximal vertical distance from the tip of the third digit (dactylion III) to the floor with the arm fully extended overhead. Participants maintained a hip angle of approximately 90° throughout the measurements. Hand span was measured as the maximal distance between the tip of the thumb (dactylion I) and the tip of the little finger (dactylion V), with the hand fully spread (Cavedon et al., 2022, 2023). A study-specific restricted set of anthropometric measurements relevant to the study outcomes was collected rather than a full ISAK profile. The anthropometrist held ISAK Level 3 certification.

#### 2.2.3 Anaerobic performance test

Anaerobic performance was assessed using a 30-s WAnT performed on an arm-crank ergometer (LODE Angio; Lode BV, Groningen, the Netherlands) with the Wingate Anaerobic Software Package (Ergometry Manager; Lode BV, Groningen, the Netherlands). The ergometer was firmly secured to a wall-mounted gymnastic ladder, and its axis of rotation was aligned with the participant’s glenohumeral joints. The protocol comprised a 2-min warm-up at 60 revolutions per minute (rpm) and 50 W, a 1-min active recovery period, and a 30-s all-out arm-cranking test against a braking torque normalized to body mass (Marszałek et al., 2019; Janssen et al., 2022). The torque factor was set at 0.55 N·m·kg^-1^ for male participants and 0.54 N·m·kg^-1^ for female participants (Lode BV, 2014). Participants were instructed to crank as rapidly as possible from the start and received strong verbal encouragement throughout the 30-s effort. Peak power (PP), mean power (MP), relative peak power (rPP), relative mean power (rMP), and fatigue slope (FS) were recorded. PP and MP were expressed in watts (W), whereas rPP and rMP were normalized to body mass and expressed in watts per kilogram (W·kg^-1^). Fatigue slope (FS) was expressed in watts per second (W·s^-1^) and represents the rate of decline in power rather than the percentage decline. FS was calculated as $FS=(PP-P_{\text{min}})/(t_{P_{\text{min}}}-t_{PP})$, where $P_{\min}$ is the lowest post-peak power, and $t_{PP}$ and $t_{P_{\text{min}}}$ are the times at which PP and $P_{\text{min}}$ occurred, respectively.

#### 2.2.4 Upper-body strength and power tests

1. Handgrip strength. Handgrip strength was assessed using a hydraulic hand dynamometer (SH5001; Saehan Corporation, Masan, Republic of Korea). Participants were seated with the tested elbow flexed to 90° and held close to the trunk. They were instructed to squeeze the dynamometer as forcefully as possible until a verbal stop signal was given. Each maximal contraction was maintained for approximately 3 s. Three maximal trials were performed with each hand, with the hands alternated between successive trials and a 1-min rest interval between trials. The dominant spiking hand was defined as the arm preferentially used for maximal spikes. For athletes capable of spiking with either arm, the arm selected for the maximal-effort spike-velocity test was used consistently for handgrip analysis. The highest value obtained with the dominant spiking hand was retained for analysis and expressed in kilograms (kg) (Jeoung, 2017).
2. 1RM bench press. The 1RM bench press test was conducted in accordance with the protocol recommended by the NSCA (Haff and Triplett, 2016). Participants lay supine on a standard bench with the head, shoulders, and buttocks remaining in contact with the bench throughout each attempt. The warm-up comprised six repetitions at approximately 60% of the estimated 1RM followed by three repetitions at approximately 80% of the estimated 1RM. Participants then completed up to five attempts to determine their individual 1RM. The load was progressively increased by 5–10% until the greatest load that could be lifted once with proper technique was identified. Rest intervals of 2 min were provided between warm-up sets and 3 min between 1RM attempts. The highest successfully lifted load was retained for analysis and expressed in kilograms (kg).
3. Seated medicine-ball chest pass. The test was conducted according to previously described procedures (Molik et al., 2013; Ahmadi et al., 2019). Participants sat on the floor with their backs against a wall, legs extended, and feet approximately 60 cm apart. Holding a 3-kg medicine ball with both hands in front of the chest and the forearms parallel to the floor, participants threw the ball straight forward as forcefully and as far as possible while keeping their backs in contact with the wall. Two maximal trials were performed, with a 45–60-s rest interval between trials (Stockbrugger and Haennel, 2001; Cavedon et al., 2022). The greatest distance was retained for analysis and recorded in centimeters (cm).

#### 2.2.5 Trunk function tests

1. Curl-up test. Participants lay supine on a mat with their knees flexed, the soles of their feet flat on the mat, their arms alongside the trunk, and their fingers fully extended on the mat (Yüksel and Sevindi, 2018). An investigator stabilized the lower limbs, or residual limb(s) in participants with lower-limb amputation, to maintain knee flexion. Participants flexed the trunk until the inferior angles of the scapulae lifted from the mat and then returned to the starting position (Cavedon et al., 2022). They completed as many valid curl-ups as possible within 30 s, and the total number of correctly completed repetitions was recorded.
2. Back-extension endurance test. The protocol was adapted from Lanning et al. (2006). Participants lay prone with the iliac crests aligned with the edge of the testing table, while the pelvis and lower limbs or residual limbs were secured with straps; manual stabilization was added when required. With the hands behind the head, participants moved from a neutral trunk position to approximately 45° of flexion and returned to neutral, completing as many repetitions as possible in 30 s. Only repetitions reaching both positions were counted. The same table, examiner, instructions, and scoring criteria were used throughout. Because this adapted procedure was not separately evaluated for reliability or validity in the present sample, its results were interpreted as exploratory.
3. Seated rotational medicine-ball throw. The protocol was adapted from previously described side medicine-ball throw procedures (Ikeda et al., 2007; Talukdar et al., 2015). Participants sat behind the starting line in a side-on position with both knees fully extended. Holding a 3-kg medicine ball with both hands in front of the abdomen, they rotated the trunk away from the throwing direction and then rapidly reversed the movement to throw the ball diagonally forward as far as possible. Three maximal trials were performed in each direction, with a 45–60-s rest interval between trials. Distance was measured from the starting line to the first point at which the ball contacted the floor. The best distance achieved in each direction was recorded in centimeters, and the mean of the two values was retained for analysis.

#### 2.2.6 Sport-specific movement tests

1. 5-m forward and backward sprint tests. The tests followed the procedures described by Cavedon et al. (2022). For the forward sprint, participants began from a stationary seated position behind the starting line and moved forward over 5 m as quickly as possible (**Figure 2A**). For the backward sprint, participants began from the same position and moved backward over 5 m as quickly as possible. The fastest time (s) obtained in each direction was retained separately for analysis. Two trials were completed in each direction.
2. Modified agility T-test. The protocol was adapted from the modified agility T-test described by Sassi et al. (2009). Participants started from a seated position behind line A and moved 5 m forward to cone B, touching its base with the right hand. They then moved 3 m laterally to the left toward cone C and touched its base with the left hand. Next, participants moved 6 m laterally to the right toward cone D and touched its base with the right hand before moving 3 m laterally back to cone B and touching its base. Finally, they moved 5 m backward as quickly as possible to cross line A (**Figure 2B**). The fastest time (s) across two trials was retained for analysis.
3. Speed-endurance test. The test was conducted according to Marszałek et al. (2015). Participants started from a seated position behind the starting line at cone A and moved back and forth as quickly as possible between cone A and cones B, C, D, E, F, and G in sequence (**Figure 2C**). They touched the base of each cone before returning to cone A and proceeding to the next cone. The fastest time (s) across two trials was retained for analysis. Completion times for all movement tests were recorded using wireless infrared timing gates (SmartSpeed; Fusion Sport, Coopers Plains, Australia) positioned at the respective start and finish lines. A 3-min recovery interval was provided between trials for all three movement tests. The infrared beams were adjusted to approximately the participant’s mid-trunk height. At the start of each trial, the center of the trunk was positioned approximately 20 cm behind the start beam to avoid premature triggering.

**Figure 2:**
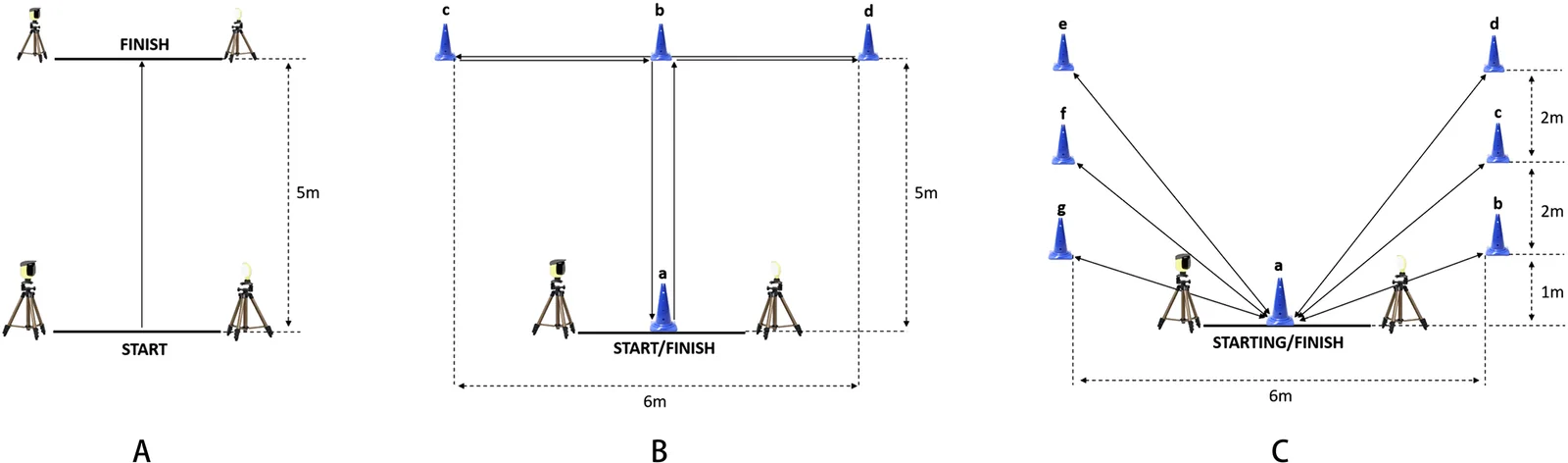
Experimental setups of sport-specific movement tests. **(A)** Layout for conducting the 5-m forward and backward sprint tests. **(B)** Layout for the modified agility T-test. **(C)** Layout for the speed-endurance test.

#### 2.2.7 Statistical analysis

Analyses were performed in R 4.4.2 (R Foundation for Statistical Computing, Vienna, Austria). Distributions were assessed using Shapiro–Wilk tests and quantile–quantile plots. Data are reported as mean ± standard deviation (SD). Exploratory between-sex comparisons used Welch’s t-tests and Hedges’ *g* with 95% confidence intervals (CIs), emphasizing effect sizes and precision. Absolute *g* values of 0.20, 0.50, and 0.80 indicated small, medium, and large effects (Cohen, 1988).

Pooled Pearson correlations and sex-adjusted partial correlations with 95% CIs assessed associations with spike velocity; sex-specific correlations were descriptive. Absolute *r* values were classified as negligible (<0.10), weak (0.10–0.39), moderate (0.40–0.69), strong (0.70–0.89), or very strong (≥0.90) (Schober et al., 2018). The Benjamini–Hochberg (BH) procedure controlled the false discovery rate (FDR) separately for the 22 between-sex comparisons, 21 pooled correlations, and 21 sex-adjusted correlations. BH-adjusted *p* < 0.05 defined statistical significance within these families.

The exploratory regression included sex (female = 0, male = 1) to account for between-sex differences, with seated vertical grip reach and seated rotational medicine-ball throw representing reach dimensions and rotational performance. Candidate domains were prespecified, but variable selection was partly informed by correlations in the same dataset used for estimation. Reporting included regression coefficients, 95% CIs, and model fit; regression *p* values were unadjusted. Diagnostics comprised predictor correlations, VIFs, leverage, and Cook’s distance. All participants were retained in the primary model; sensitivity analyses excluded the observation with the largest Cook’s distance and used 5,000 bootstrap resamples to obtain percentile 95% CIs. These analyses assessed sensitivity within this sample and did not establish that selection-related optimism had been removed. Sex-adjusted correlations and regression were also repeated in VS1 athletes (*n* = 27), with BH correction applied separately to the 21 correlations (**Supplementary Tables S1**, **S2**).

## 3 Results

### 3.1 Between-sex comparisons

**Table 1** summarizes the exploratory between-sex comparisons; all *p* values in this paragraph are BH-adjusted. Men achieved higher spike velocity than women (67.22 ± 6.74 vs. 51.51 ± 6.04 km/h; Hedges’ *g* = 2.38, 95% CI [1.43, 3.30], *p* < 0.001). Sitting height, seated vertical grip reach, hand span, and BMI were also higher in men (*g* = 0.86–2.41; all *p* ≤ 0.025). Men had higher PP, MP, rPP, and fatigue slope (all *p* ≤ 0.046); the estimate for rMP was less precise (*g* = 0.52, 95% CI [−0.20, 1.22], *p* = 0.168). Large differences favored men for handgrip strength, 1RM bench press, chest-pass distance, and mean rotational-throw distance (*g* = 1.86–3.76; all *p* < 0.001). Women completed more back-extension repetitions (*g* = −0.92, 95% CI [−1.65, −0.18], *p* = 0.019), whereas the curl-up estimate was uncertain (*g* = −0.39, 95% CI [−1.09, 0.32], *p* = 0.283). Men recorded shorter forward and backward 5-m sprint times (*g* = −1.16 and −1.13; *p* = 0.006 and 0.007, respectively). Estimates for the modified agility T-test and speed-endurance test were less precise (*g* = −0.51 and −0.50; *p* = 0.173 and 0.198, respectively). These exploratory comparisons describe the sample and provide context for interpreting the pooled correlations.

**Table 1:** Descriptive statistics and between-sex comparisons of spike velocity and anthropometric, anaerobic, and physical fitness characteristics in elite sitting volleyball players.

Variables	Total (*n* = 30) Mean ± *SD*	Male (*n* = 16) Mean ± *SD*	Female (*n* = 14) Mean ± *SD*	Hedges’ *g* [95% CI]	BH-adjusted *p*
Spike velocity (km/h)	59.89 ± 10.17	67.22 ± 6.74	51.51 ± 6.04	2.38 [1.43, 3.30]	<0.001
Anthropometric characteristics					
Sitting height (cm)	93.56 ± 5.04	97.10 ± 3.97	89.51 ± 2.31	2.23 [1.31, 3.13]	<0.001
Seated vertical grip reach (cm)	144.70 ± 10.48	152.30 ± 6.91	136.01 ± 6.15	2.41 [1.46, 3.34]	<0.001
Hand span (cm)	18.84 ± 1.38	19.52 ± 1.28	18.06 ± 1.07	1.20 [0.43, 1.96]	0.004
BMI (kg/m²)	22.18 ± 3.15	23.38 ± 3.62	20.81 ± 1.79	0.86 [0.12, 1.58]	0.025
Anaerobic performance					
PP (W)	462.84 ± 154.60	575.00 ± 116.23	334.64 ± 67.45	2.42 [1.47, 3.35]	<0.001
MP (W)	277.58 ± 81.52	330.07 ± 75.67	217.58 ± 30.80	1.85 [0.99, 2.68]	<0.001
rPP (W·kg^-1^)	6.65 ± 1.79	7.41 ± 1.91	5.78 ± 1.18	0.98 [0.23, 1.72]	0.013
rMP (W·kg^-1^)	4.02 ± 0.91	4.24 ± 1.09	3.76 ± 0.60	0.52 [−0.20, 1.22]	0.168
FS (W·s^-1^)	10.41 ± 6.82	12.74 ± 8.12	7.75 ± 3.67	0.75 [0.02, 1.47]	0.046
Upper-body strength and power					
Handgrip strength (kg)	40.88 ± 9.96	49.02 ± 5.81	31.59 ± 2.80	3.64 [2.45, 4.80]	<0.001
1RM bench press (kg)	74.85 ± 22.45	93.31 ± 12.96	53.75 ± 5.61	3.76 [2.55, 4.95]	<0.001
Seated medicine-ball chest pass (cm)	580.80 ± 60.47	621.19 ± 49.16	534.64 ± 32.66	1.99 [1.11, 2.85]	<0.001
Trunk function					
Curl-up test (repetitions)	37.10 ± 4.57	36.25 ± 4.64	38.07 ± 4.46	−0.39 [−1.09, 0.32]	0.283
Back-extension endurance test (repetitions)	47.30 ± 6.78	44.56 ± 6.69	50.43 ± 5.57	−0.92 [−1.65, −0.18]	0.019
Seated rotational medicine-ball throw (mean) (cm)	693.78 ± 120.83	771.78 ± 103.92	604.64 ± 63.72	1.86 [1.00, 2.69]	<0.001
Seated rotational medicine-ball throw (left) (cm)	692.27 ± 132.16	779.31 ± 117.72	592.79 ± 54.71	1.93 [1.06, 2.81]	<0.001
Seated rotational medicine-ball throw (right) (cm)	695.30 ± 119.07	764.25 ± 97.18	616.50 ± 90.50	1.53 [0.71, 2.35]	<0.001
Sport-specific movement performance					
5-m forward sprint test (s)	2.13 ± 0.37	1.95 ± 0.29	2.33 ± 0.36	−1.16 [−1.91, −0.39]	0.006
5-m backward sprint test (s)	2.10 ± 0.54	1.85 ± 0.40	2.39 ± 0.53	−1.13 [−1.88, −0.36]	0.007
Modified agility T-test (s)	9.27 ± 1.18	8.99 ± 1.31	9.59 ± 0.94	−0.51 [−1.21, 0.21]	0.173
Speed-endurance test (s)	21.77 ± 2.33	21.22 ± 1.61	22.41 ± 2.88	−0.50 [−1.21, 0.21]	0.198

Between-sex comparisons were exploratory. Welch-test p values for spike velocity and 21 predictors were adjusted together using the Benjamini–Hochberg procedure; interpretation emphasizes Hedges’ g and its 95% CI. Positive g indicates higher values in men; for timed movement tests, lower values indicate faster performance. SD, standard deviation; CI, confidence interval; BMI, body mass index; PP, peak power; MP, mean power; rPP, relative peak power; rMP, relative mean power; FS, fatigue slope; 1RM, one-repetition maximum; BH, Benjamini–Hochberg.

### 3.2 Correlations with spike velocity

Across the six spike attempts, the within-session ICC was 0.89 and the coefficient of variation was 17.18%. **Table 2** presents the pooled, sex-adjusted, and sex-specific correlations with spike velocity. In the pooled sample, strong positive correlations remained significant after Benjamini–Hochberg correction for seated vertical grip reach (*r* = 0.80), sitting height (*r* = 0.76), handgrip strength (*r* = 0.75), 1RM bench press (*r* = 0.74), and seated medicine-ball chest-pass distance (*r* = 0.70), while moderate positive correlations were observed for hand span (*r* = 0.69), seated rotational medicine-ball throw (*r* = 0.65), PP (*r* = 0.51), and MP (*r* = 0.51). After adjustment for sex, however, all of these associations were attenuated and none remained statistically significant after Benjamini–Hochberg correction. The largest sex-adjusted partial correlations were observed for hand span (partial *r* = 0.52, 95% CI [0.19, 0.74], raw *p* = 0.004, adjusted *p* = 0.057), seated vertical grip reach (partial *r* = 0.48 [0.14, 0.72], raw *p* = 0.009, adjusted *p* = 0.057), rPP (partial *r* = −0.48 [−0.72, −0.13], raw *p* = 0.009, adjusted *p* = 0.057), and sitting height (partial *r* = 0.40 [0.04, 0.67], raw *p* = 0.031, adjusted *p* = 0.148). The sex-specific descriptive analyses showed positive correlations for sitting height, seated vertical grip reach, and hand span in both men (*r* = 0.34–0.47) and women (*r* = 0.56–0.68), whereas several strength and absolute Wingate associations were weaker or less consistent across sexes. These subgroup estimates were interpreted descriptively because of the small sample sizes. As illustrated in **Figure 3**, several strong or moderate pooled relationships were less apparent within the male and female subgroups, particularly for peak power and one-repetition maximum bench press.

**Table 2:** Pooled, sex-adjusted, and sex-specific correlations of anthropometric, anaerobic, and physical fitness characteristics with spike velocity in elite sitting volleyball players.

Variables	Pooled Pearson’s *r* [95% CI]	*p*	BH-adjusted *p*	Sex-adjusted partial *r* [95% CI]	*p*	BH-adjusted *p*	Men Pearson’s *r* [95% CI]	Women Pearson’s *r* [95% CI]
Spike velocity (km/h)	—	—	—	—	—	—	—	—
Anthropometric characteristics								
Sitting height (cm)	0.76 [0.55, 0.88]	<0.001	<0.001	0.40 [0.04, 0.67]	0.031	0.148	0.34 [−0.19, 0.72]	0.56 [0.04, 0.84]
Seated vertical grip reach (cm)	0.80 [0.62, 0.90]	<0.001	<0.001	0.48 [0.14, 0.72]	0.009	0.057	0.34 [−0.19, 0.72]	0.68 [0.23, 0.89]
Hand span (cm)	0.69 [0.44, 0.84]	<0.001	<0.001	0.52 [0.19, 0.74]	0.004	0.057	0.47 [−0.04, 0.78]	0.61 [0.11, 0.86]
BMI (kg/m²)	0.40 [0.05, 0.67]	0.027	0.051	0.14 [−0.24, 0.48]	0.472	0.640	0.29 [−0.24, 0.69]	−0.25 [−0.69, 0.33]
Anaerobic performance								
PP (W)	0.51 [0.19, 0.74]	0.004	0.009	−0.27 [−0.58, 0.11]	0.155	0.343	−0.38 [−0.74, 0.14]	−0.04 [−0.56, 0.50]
MP (W)	0.51 [0.18, 0.73]	0.004	0.009	−0.09 [−0.44, 0.29]	0.643	0.764	−0.14 [−0.60, 0.38]	0.05 [−0.49, 0.57]
rPP (W·kg^-1^)	0.10 [−0.27, 0.45]	0.595	0.665	−0.48 [−0.72, −0.13]	0.009	0.057	−0.59 [−0.84, −0.13]	−0.26 [−0.70, 0.31]
rMP (W·kg^-1^)	0.00 [−0.36, 0.36]	0.995	0.995	−0.34 [−0.63, 0.03]	0.067	0.256	−0.41 [−0.75, 0.11]	−0.22 [−0.67, 0.36]
FS (W·s^-1^)	0.31 [−0.06, 0.60]	0.099	0.145	0.03 [−0.34, 0.39]	0.889	0.938	−0.02 [−0.51, 0.48]	0.16 [−0.41, 0.63]
Upper-body strength and power								
Handgrip strength (kg)	0.75 [0.53, 0.87]	<0.001	<0.001	0.18 [−0.20, 0.51]	0.360	0.570	0.38 [−0.14, 0.74]	−0.35 [−0.75, 0.22]
1RM bench press (kg)	0.74 [0.53, 0.87]	<0.001	<0.001	0.16 [−0.22, 0.50]	0.417	0.609	0.25 [−0.29, 0.66]	−0.07 [−0.58, 0.48]
Seated medicine-ball chest pass (cm)	0.70 [0.45, 0.85]	<0.001	<0.001	0.30 [−0.07, 0.60]	0.113	0.306	0.31 [−0.22, 0.70]	0.29 [−0.28, 0.71]
Trunk function								
Curl-up test (repetitions)	−0.29 [−0.59, 0.08]	0.117	0.159	−0.22 [−0.54, 0.16]	0.250	0.433	−0.27 [−0.67, 0.26]	−0.16 [−0.64, 0.41]
Back-extension endurance test (repetitions)	−0.38 [−0.65, −0.03]	0.037	0.058	−0.07 [−0.43, 0.31]	0.721	0.806	−0.37 [−0.73, 0.15]	0.40 [−0.17, 0.77]
Seated rotational medicine-ball throw (mean) (cm)	0.65 [0.38, 0.82]	<0.001	<0.001	0.23 [−0.15, 0.55]	0.223	0.424	0.24 [−0.29, 0.66]	0.24 [−0.33, 0.68]
Seated rotational medicine-ball throw (left) (cm)	0.70 [0.46, 0.85]	<0.001	<0.001	0.33 [−0.04, 0.62]	0.082	0.285	0.33 [−0.20, 0.71]	0.39 [−0.18, 0.76]
Seated rotational medicine-ball throw (right) (cm)	0.54 [0.23, 0.76]	0.002	0.004	0.11 [−0.27, 0.46]	0.583	0.720	0.11 [−0.41, 0.57]	0.10 [−0.45, 0.60]
Sport-specific movement performance								
5-m forward sprint test (s)	−0.24 [−0.56, 0.13]	0.194	0.245	0.32 [−0.06, 0.61]	0.095	0.301	0.38 [−0.14, 0.74]	0.26 [−0.32, 0.69]
5-m backward sprint test (s)	−0.40 [−0.66, −0.04]	0.030	0.052	0.01 [−0.36, 0.38]	0.952	0.952	0.25 [−0.28, 0.67]	−0.22 [−0.67, 0.35]
Modified agility T-test (s)	−0.04 [−0.40, 0.32]	0.819	0.864	0.27 [−0.11, 0.58]	0.162	0.343	0.39 [−0.13, 0.74]	0.05 [−0.50, 0.56]
Speed-endurance test (s)	−0.13 [−0.47, 0.24]	0.503	0.597	0.12 [−0.25, 0.47]	0.520	0.659	0.22 [−0.31, 0.65]	0.07 [−0.48, 0.58]

Pooled Pearson correlations and sex-adjusted partial correlations are reported with nominal 95% CIs, without multiplicity adjustment of the intervals. Raw and BH-adjusted *p* values are shown; BH correction was applied separately to the 21 pooled and 21 sex-adjusted correlations. Sex-specific correlations are descriptive. BMI, body mass index; CI, confidence interval; BH, Benjamini–Hochberg; PP, peak power; MP, mean power; rPP, relative peak power; rMP, relative mean power; FS, fatigue slope; 1RM, one-repetition maximum.

**Figure 3:**
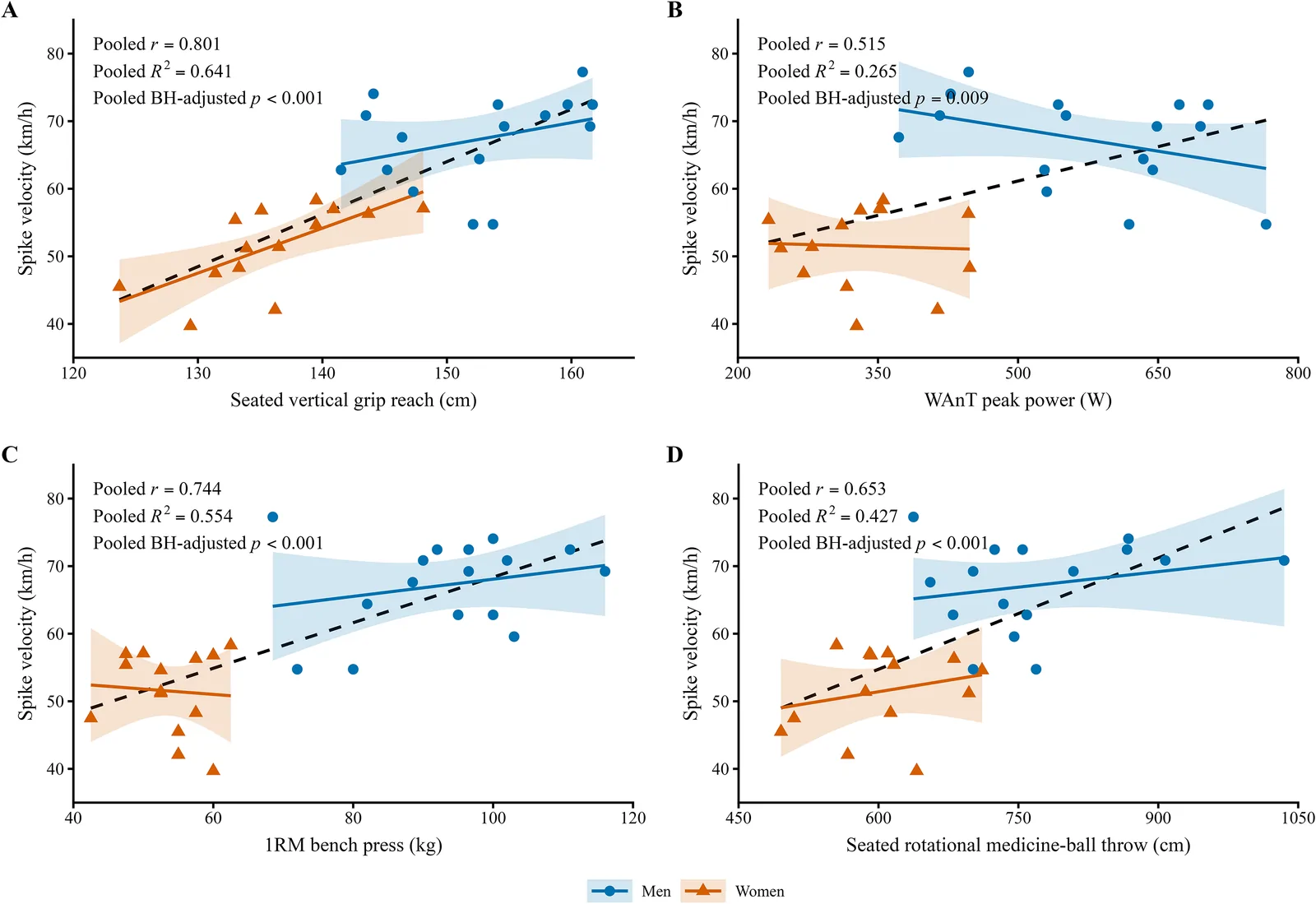
Bivariate associations between spike velocity and selected anthropometric and physical-performance variables: **(A)** seated vertical grip reach, **(B)** Wingate peak power, **(C)** one-repetition maximum (1RM) bench press, and **(D)** seated rotational medicine-ball throw. Male and female players are shown using different symbols and colours. Solid lines show sex-specific fits (shading: 95% confidence intervals); the dashed black line shows the pooled fit. Panels report pooled Pearson’s *r*, *R*², and Benjamini–Hochberg (BH)-adjusted *p* values; correction covers all 21 correlations in **Table 2**.

### 3.3 Exploratory multiple linear regression analysis

The exploratory regression model included sex, seated vertical grip reach, and seated rotational medicine-ball throw (**Table 3**). The model explained 71.9% of the variance in spike velocity (*R²* = 0.719, adjusted *R²* = 0.687, *F*(3, 26) = 22.21, *p* < 0.001). After simultaneous adjustment, seated vertical grip reach retained a positive coefficient (*B* = 0.457, 95% CI [0.120, 0.794], *β* = 0.471, *t* = 2.79, *p* = 0.010), whereas the coefficients for sex (*B* = 5.732, 95% CI [−2.242, 13.707], *β* = 0.286, *p* = 0.152) and seated rotational medicine-ball throw (*B* = 0.015, 95% CI [−0.010, 0.040], *β* = 0.180, *p* = 0.230) were imprecise. Correlations among the three predictors were *r* = 0.79 for sex with seated vertical grip reach, *r* = 0.70 for sex with seated rotational medicine-ball throw, and *r* = 0.58 for seated vertical grip reach with seated rotational medicine-ball throw. Variance inflation factors ranged from 1.98 to 3.47. The maximum leverage value was 0.402 (case 16), whereas the largest Cook’s distance was 0.182 (case 18). After excluding case 18, the model estimates were similar (*R²* = 0.718, adjusted *R²* = 0.684); seated vertical grip reach remained positively associated with spike velocity (*B* = 0.377, 95% CI [0.037, 0.718], *p* = 0.031), while sex (*p* = 0.177) and rotational throw (*p* = 0.093) remained uncertain. In 5,000 bootstrap resamples, the percentile 95% confidence intervals were [−1.29, 10.65] for sex, [0.18, 0.77] for seated vertical grip reach, and [−0.008, 0.043] for rotational throw. Thus, the direction of the principal seated-reach finding was not materially altered by removal of the most influential case, although the model remains exploratory and requires validation in a larger independent sample.

**Table 3:** Exploratory sex-adjusted multiple linear regression model for spike velocity in elite sitting volleyball players.

Predictor	*B* [95% CI]	*SE*	*β*	*t*	*p*	VIF
Sex (male vs. female)	5.732 [−2.242, 13.707]	3.879	0.286	1.48	0.152	3.473
Seated vertical grip reach	0.457 [0.120, 0.794]	0.164	0.471	2.79	0.010	2.649
Seated rotational medicine-ball throw	0.015 [−0.010, 0.040]	0.012	0.180	1.23	0.230	1.978

Overall model: *R²* = 0.719, adjusted *R²* = 0.687, *F*(3, 26) = 22.21, *p* < 0.001. Sex was coded female = 0 and male = 1. The model was explicitly exploratory and was not intended as a validated predictive model. *B*, unstandardized regression coefficient; *β*, standardized regression coefficient; CI, confidence interval; *SE*, standard error; VIF, variance inflation factor.

As a sensitivity analysis, the three VS2 athletes were excluded and the sex-adjusted correlations and regression were re-estimated in the 27 VS1 athletes. Across the 21 sex-adjusted correlations, the median absolute change in partial *r* was approximately 0.07, and no association was significant after Benjamini–Hochberg correction in the VS1-only sample. The VS1-only regression showed similar model fit (*R*² = 0.765, adjusted *R*² = 0.735), and seated vertical grip reach retained a positive coefficient (*B* = 0.506, 95% CI [0.141, 0.872], *p* = 0.009), whereas the sex and rotational-throw coefficients remained imprecise (**Supplementary Tables S1**, **S2**).

## 4 Discussion

This study examined physical correlates of spike velocity in elite sitting volleyball players. Several anthropometric, strength, and absolute anaerobic-power measures were positively associated with spike velocity in the pooled sample. After adjustment for sex, most associations weakened substantially and none remained significant after FDR correction. Seated anthropometric measures showed the most consistent positive pattern across analyses, with seated vertical grip reach retaining a positive coefficient in the exploratory sex-adjusted regression. These findings suggest that the strong pooled relationships partly reflected between-sex differences, while the adjusted estimates remained too imprecise to establish independent physical determinants of spike velocity. The differing patterns across tests also highlight the need to interpret general strength, arm-crank power, and movement performance in relation to the specific demands of a single maximal spike.

### 4.1 Sex-related differences and interpretation of pooled associations

The large sex-related difference in spike velocity was accompanied by marked differences in seated anthropometry, absolute anaerobic power, upper-body strength, medicine-ball throwing performance, and short-distance movement speed. This pattern is consistent with previous sitting volleyball studies reporting sex-related differences in strength-, power-, and speed-oriented tests (Ahmadi et al., 2019) and distinct physical profiles between national- and league-level players (Ahmadi et al., 2020). In standing volleyball, elite male and female players also differ in spike-jump mechanics and the distribution of arm-swing techniques, reflecting differences in anthropometry, force production, and movement strategy (Fuchs et al., 2019; Giatsis and Tilp, 2022). The revised analyses demonstrate the importance of this separation by sex: many strong pooled correlations were markedly reduced after sex was controlled statistically. None of the sex-adjusted correlations remained significant after FDR correction, although seated vertical grip reach, hand span, and sitting height retained moderate positive effect estimates. The descriptive within-sex analyses showed positive anthropometric correlations in both men and women, whereas several strength and anaerobic-power relationships were less consistent. Thus, pooled associations describe meaningful differences across the mixed-sex elite sample but should not be interpreted as equivalent within-sex relationships. This pattern is also visually apparent in the sex-specific distributions and fitted lines shown in **Figure 3**. The substantial between-athlete variation in this mixed-sex sample may also partly explain the high within-session ICC (0.89) despite appreciable trial-to-trial variation (CV = 17.18%).

### 4.2 Seated reach as the primary anthropometric correlate

Among the anthropometric variables, seated vertical grip reach showed the strongest pooled association with spike velocity and retained a moderate positive association after adjustment for sex (partial *r* = 0.48), although its FDR-adjusted *p* value was 0.057. Positive within-sex correlations were also observed in men (*r* = 0.34) and women (*r* = 0.68). In the exploratory regression, seated vertical grip reach retained a positive coefficient after adjustment for sex and rotational throwing performance, with a positive coefficient after exclusion of the observation with the largest Cook’s distance and a bootstrap 95% CI excluding zero. The positive direction was consistent across these analyses, although the adjusted estimate remained imprecise. Seated vertical grip reach is a composite measure influenced by sitting height, upper-limb length, and overhead reach; greater reach may allow a higher contact position and a longer effective acceleration path before impact. This interpretation is consistent with sitting volleyball serve research showing that vertical reach contributed to ball-impact height and that impact height contributed to serve velocity (Cavedon et al., 2023), as well as earlier associations among reach measures, spike-position height, field-test performance, and game performance (Marszałek et al., 2015; Molik et al., 2017). Tennis research further indicates that height, sitting height, arm dimensions, and contact height are associated with serve velocity in several populations (Bonato et al., 2015; Fett et al., 2020; Hayes et al., 2021; Sánchez-Pay et al., 2021; Baiget et al., 2023). Nevertheless, the present adjusted estimates remain imprecise, and contact height, hand velocity, and upper-limb segment lengths were not measured directly; therefore, the findings identify seated reach as an association rather than establish a biomechanical mechanism.

### 4.3 Upper-body strength and anaerobic power

Handgrip strength, 1RM bench press, and seated medicine-ball chest-pass distance were strongly associated with spike velocity in the pooled sample, but these associations were substantially weaker after adjustment for sex. The sex-adjusted partial correlations were 0.18 for handgrip strength, 0.16 for 1RM bench press, and 0.30 for chest-pass distance, and none remained significant after FDR correction. The within-sex estimates were also less consistent for handgrip strength (men *r* = 0.38; women *r* = −0.35) and 1RM bench press (men *r* = 0.25; women *r* = −0.07), whereas chest-pass correlations were similar in direction and magnitude in men (*r* = 0.31) and women (*r* = 0.29). These findings suggest that the strong pooled strength relationships were influenced substantially by between-sex differences. Evidence from standing volleyball and team handball nevertheless supports a broader association between upper-body force-generating capacity and high-velocity overarm actions (Forthomme et al., 2005; Gorostiaga et al., 2005; Marques et al., 2007; Baena-Raya et al., 2021). Within sitting volleyball, Fashi et al. (2025) documented greater internal- than external-rotator torque, work, and power in the dominant shoulder of elite male players at 60°·s^-1^. Their study provides a joint-specific strength profile, but neither those controlled-velocity measures nor our handgrip and 1RM bench-press tests directly assess rapid shoulder internal rotation during a spike. The attenuation of our general-strength associations after sex adjustment therefore does not exclude a contribution from task-specific shoulder function. General strength tests may therefore complement assessments that more directly capture the rapid force production and coordination required during a spike.

Absolute PP and MP were moderately positively correlated with spike velocity in the pooled analysis. After adjustment for sex, estimates were negative and imprecise (PP: partial *r* = −0.27, 95% CI [−0.58, 0.11]; MP: partial *r* = −0.09, 95% CI [−0.44, 0.29]). Their within-sex correlations were also not consistently positive. This pattern indicates that the pooled associations with absolute Wingate power were strongly influenced by between-sex differences in both power output and spike velocity. Relative peak power showed an inverse sex-adjusted estimate (partial *r* = −0.48) and negative within-sex correlations in both men and women, but the FDR-adjusted *p* value was 0.057 and the subgroup estimates were based on small samples. The uncertainty in these estimates limits physiological interpretation. The arm-crank WAnT and spike also differ substantially in task demands: the former is a bilateral, cyclical, 30-s test, whereas the latter is a brief, unilateral, noncyclical action requiring precise segmental timing. Overall, the analyses do not support a clear sex-independent association between absolute Wingate power and maximal spike velocity in this sample.

### 4.4 Trunk function and sport-specific movement performance

The associations with spike velocity differed according to the trunk test. Seated rotational medicine-ball throw distance showed a moderate positive pooled correlation. Curl-up and back-extension estimates were negative (*r* = −0.29, 95% CI [−0.59, 0.08], and *r* = −0.38, 95% CI [−0.65, −0.03], respectively), but neither met the FDR-adjusted significance threshold. After adjustment for sex, the rotational-throw association was attenuated to partial *r* = 0.23, with similar small positive within-sex correlations in men and women (both *r* = 0.24). The rotational throw also did not show a clear independent coefficient in the exploratory regression (*B* = 0.015, *p* = 0.230), although the estimate increased modestly after removal of the observation with the largest Cook’s distance. The rotational throw requires rapid trunk rotation coordinated with arm movement and therefore shares more mechanical features with an overhand strike than repetition-based endurance tests (Ikeda et al., 2007; Talukdar et al., 2015; Rodriguez-Perea et al., 2024). The attenuation after adjustment suggests that the pooled relationship partly overlaps with sex and seated anthropometric differences. The pooled correlation was stronger for the left than the right rotational throw (*r* = 0.70 vs. 0.54), whereas the corresponding sex-adjusted correlations were weaker (partial *r* = 0.33 vs. 0.11). This directional difference is descriptive: the two correlations were not formally compared, and the present data cannot establish whether it reflects technique, functional laterality, or sampling variation.

Sex-adjusted associations between sport-specific movement measures and spike velocity were small to moderate (partial *r* = 0.01–0.32), with all 95% CIs spanning zero. Several estimates changed direction after adjustment, and their uncertainty limits conclusions about the magnitude and direction of these relationships. Previous studies have demonstrated relationships between WAnT outcomes and sport-specific movement tests (Marszałek et al., 2015), between field fitness and coach-rated sitting volleyball performance (Jeoung, 2017), and between anthropometric and strength characteristics and floor-based sprint performance (Cavedon et al., 2022). These tests therefore remain relevant to broader sitting-volleyball performance, but the present data do not show a consistent relationship with the velocity of a single standardized maximal spike. Field-movement tests and direct spike-velocity assessment may therefore capture complementary aspects of performance.

### 4.5 Limitations and future directions

The cross-sectional design precludes causal inference. The sample (*n* = 30) fell below the estimated requirement of 35 for a single bivariate correlation, limiting precision, particularly in sex-specific analyses. That calculation did not establish adequate power for multiple comparisons or regression. Because predictor selection was partly informed by observed correlations, model fit may be optimistic despite sex adjustment and sensitivity analyses; the regression remains exploratory and requires independent validation. In addition, sex coincided with membership of the men’s or women’s national team, so sex-related and team-related influences could not be distinguished. The sport-class imbalance (27 VS1 and three VS2 athletes) limited class-specific analysis and generalization to VS2 athletes, despite similar results in the VS1-only sensitivity analysis. This imbalance is particularly relevant given ongoing efforts to develop evidence-based, sport-specific classification and previous research examining skill performance across functional sport classes (Esatbeyoglu et al., 2021; Marszałek et al., 2024). Impairment heterogeneity also limits the comparability of conventional BMI, particularly in athletes with lower-limb amputation. Methodologically, set height and position and radar-alignment angles were not quantified. The reported within-session ICC and CV do not quantify setup-specific radar measurement error or establish between-session reproducibility of the best-of-six score. Contact height, segmental kinematics, and impact mechanics were not measured, so biomechanical explanations remain tentative. Larger multicenter studies with adequate representation of both sexes and sport classes, direct biomechanical measurements, and longitudinal follow-up are needed.

## 5 Conclusion

In this mixed-sex sample of elite sitting volleyball players, several seated anthropometric, strength, medicine-ball, and absolute anaerobic-power measures showed strong or moderate pooled associations with spike velocity. However, most of these relationships were substantially attenuated after accounting for sex, and no sex-adjusted correlation remained significant after FDR correction. Seated anthropometric measures, particularly seated vertical grip reach and hand span, showed the most consistent positive pattern across pooled, sex-adjusted, and sex-specific analyses. In an explicitly exploratory regression including sex, seated vertical grip reach, and rotational throwing performance, seated vertical grip reach retained a positive coefficient and the direction of this finding was similar after removal of the most influential case. These findings highlight the potential value of seated anthropometric and physical-performance assessments as complementary tools for understanding spike-velocity profiles in elite sitting volleyball players. When considered alongside direct technical assessment, they may contribute to more comprehensive athlete profiling and individualized performance evaluation.

## Data Availability

The raw data supporting the conclusions of this article will be made available by the authors, without undue reservation.
