# Supplementary material for: Associations between spike velocity and physical characteristics in elite sitting volleyball players
Source: Front Physiol. 2026 Oct 12;17:1992570. doi: 10.3389/fphys.2026.1992570 (PMC13696302; doi:10.3389/fphys.2026.1992570)
Supplement: Supplementary file 1 [file DataSheet1.pdf]

Supplementary Table S1 | Sex-adjusted partial correlations with spike velocity in the full and VS1-only cohorts

Variables	Full cohort partial *r* [95% CI]	Raw *p*	BH-adjusted *p*	VS1-only partial *r* [95% CI]	Raw *p*	BH-adjusted *p*
Spike velocity (km/h)	—	—	—	—	—	—
**Anthropometric characteristics**						
Sitting height (cm)	0.40 [0.04, 0.67]	0.031	0.148	0.44 [0.07, 0.71]	0.023	0.162
Seated vertical grip reach (cm)	0.48 [0.14, 0.72]	0.009	0.057	0.52 [0.17, 0.76]	0.006	0.063
Hand span (cm)	0.52 [0.19, 0.74]	0.004	0.057	0.56 [0.22, 0.78]	0.003	0.057
BMI (kg/m²)	0.14 [−0.24, 0.48]	0.472	0.640	0.04 [−0.35, 0.42]	0.846	0.987
**Anaerobic performance**						
PP (W)	−0.27 [−0.58, 0.11]	0.155	0.343	−0.28 [−0.60, 0.12]	0.168	0.464
MP (W)	−0.09 [−0.44, 0.29]	0.643	0.764	−0.07 [−0.45, 0.33]	0.733	0.906
rPP ($W \cdot kg^{-1}$)	−0.48 [−0.72, −0.13]	0.009	0.057	−0.42 [−0.70, −0.04]	0.031	0.162
rMP ($W \cdot kg^{-1}$)	−0.34 [−0.63, 0.03]	0.067	0.256	−0.26 [−0.59, 0.14]	0.199	0.464
FS ($W \cdot s^{-1}$)	0.03 [−0.34, 0.39]	0.889	0.938	−0.15 [−0.51, 0.25]	0.458	0.641
**Upper-body strength and power**						
Handgrip strength (kg)	0.18 [−0.20, 0.51]	0.360	0.570	0.27 [−0.13, 0.60]	0.174	0.464
1RM bench press (kg)	0.16 [−0.22, 0.49]	0.417	0.609	0.00 [−0.39, 0.39]	0.999	0.999
Seated medicine-ball chest pass (cm)	0.30 [−0.07, 0.60]	0.113	0.306	0.19 [−0.21, 0.54]	0.353	0.641
**Trunk function**						
Curl-up test (repetitions)	−0.22 [−0.54, 0.16]	0.250	0.433	−0.16 [−0.51, 0.25]	0.444	0.641
Back-extension endurance test (repetitions)	−0.07 [−0.43, 0.30]	0.721	0.806	0.00 [−0.38, 0.39]	0.984	0.999
Seated rotational medicine-ball throw (mean) (cm)	0.23 [−0.15, 0.55]	0.223	0.424	0.20 [−0.20, 0.55]	0.324	0.641
Seated rotational medicine-ball throw (left) (cm)	0.33 [−0.04, 0.62]	0.082	0.285	0.28 [−0.12, 0.60]	0.164	0.464
Seated rotational medicine-ball throw (right) (cm)	0.11 [−0.27, 0.46]	0.583	0.720	0.10 [−0.30, 0.47]	0.638	0.837
**Sport-specific movement performance**						
5-m forward sprint test (s)	0.32 [−0.06, 0.61]	0.095	0.301	0.27 [−0.13, 0.59]	0.187	0.464
5-m backward sprint test (s)	0.01 [−0.36, 0.38]	0.952	0.952	−0.18 [−0.53, 0.22]	0.379	0.641
Modified agility T-test (s)	0.27 [−0.11, 0.58]	0.162	0.343	0.15 [−0.25, 0.51]	0.452	0.641
Speed-endurance test (s)	0.12 [−0.25, 0.47]	0.520	0.659	−0.01 [−0.40, 0.38]	0.947	0.999

Note. Sex-adjusted partial Pearson correlations with spike velocity are shown for the full cohort (n = 30) and the VS1-only cohort (n = 27). The Benjamini–Hochberg (BH) procedure controlled the false discovery rate (FDR) separately across 21 tests per cohort; the 95% confidence intervals (CIs) were not adjusted. VS1 and VS2 are World ParaVolley sport classes. Relative power was normalized to net body mass (excluding prostheses). The mean rotational throw is the average of the best distances in each direction. Lower times indicate faster movement. Abbreviations: r, correlation coefficient; BMI, body mass index; FS, fatigue slope; MP, mean power; PP, peak power; rMP, relative mean power; rPP, relative peak power; 1RM, one-repetition maximum.

Supplementary Table S2 | Multiple regression sensitivity analysis after exclusion of three VS2 athletes

Predictor	Full cohort *B* [95% CI]	*β*	*p*	VS1-only *B* [95% CI]	*β*	*p*
Sex (male vs. female)	5.732 [−2.242, 13.707]	0.286	0.152	6.859 [−1.382, 15.100]	0.332	0.099
Seated vertical grip reach (cm)	0.457 [0.120, 0.794]	0.471	0.010	0.506 [0.141, 0.872]	0.498	0.009
Seated rotational medicine-ball throw (mean, cm)	0.015 [−0.010, 0.040]	0.180	0.230	0.010 [−0.016, 0.035]	0.114	0.440

Note. The outcome in both three-predictor models is spike velocity (km/h). Sex was coded female = 0 and male = 1. B is the unstandardized regression coefficient: for sex, the adjusted male–female difference in km/h; for the continuous predictors, the change in km/h per 1 cm increase. β is the standardized regression coefficient. The 95% confidence intervals (CIs) are nominal, and regression p values are unadjusted for multiple comparisons. Abbreviations: R^2, coefficient of determination; VS1 and VS2, World ParaVolley sport classes.

Model fit. Full cohort (n = 30): $R^2 = 0.719$, adjusted $R^2 = 0.687$, $F(3, 26) = 22.21$, $p < 0.001$. VS1-only cohort (n = 27): $R^2 = 0.765$, adjusted $R^2 = 0.735$, $F(3, 23) = 25.01$, $p < 0.001$.
